# Proteomics Evaluation of Semen of Clinically Healthy Beagle-Breed Dogs

**DOI:** 10.3390/vetsci9120697

**Published:** 2022-12-15

**Authors:** Pagona G. Gouletsou, George Th. Tsangaris, Eleni I. Katsarou, Maria V. Bourganou, Mariana S. Barbagianni, Athina P. Venianaki, Efterpi Bouroutzika, Athanasios K. Anagnostopoulos, George C. Fthenakis, Angeliki I. Katsafadou

**Affiliations:** 1 Veterinary Faculty, University of Thessaly, 43100 Karditsa, Greece; 2 Proteomics Research Unit, Biomedical Research Foundation of the Academy of Athens, 11527 Athens, Greece; 3 Faculty of Public and One Health, University of Thessaly, 43100 Karditsa, Greece

**Keywords:** diagnosis, dog, LC-MS/MS, LTQ Orbitrap Elite, proteomics, reference values, semen

## Abstract

**Simple Summary:**

Proteomics aims to identify proteins present in a sample and to study their expression during various physiological or pathological conditions. The proteome includes all proteins present in a cell or a tissue at any given time; this takes into account all post-translational modifications that occur, and is highly dynamic. The present work used high-throughput technologies to study the proteome of the semen of dogs. In total, 42 proteins were identified in the semen sperm-rich fraction and 43 proteins in the semen prostatic fraction. In general, the proteins identified are involved mostly in supporting spermatozoan maturation, survival and motility, enhancing the reproductive performance of male animals. Future work can focus on the quantification of proteins identified in semen and compare findings with results in samples from animals with suboptimal fertility. The findings can provide a potential for proteomics examination of semen as a tool in se-men evaluation. This can be particularly useful in stud animals, also given its advantage as a non-invasive method.

**Abstract:**

The objectives of the present work were to evaluate the semen of dogs by means of proteomics methods and to compare with proteomics results of the blood of the animals, in order to increase available knowledge on the topic and present relevant reference values for semen samples. Semen samples were collected from five Beagle-breed dogs. Reproductive assessment of the animals by means of clinical, ultrasonographic and seminological examinations confirmed their reproductive health. The sperm-rich fraction and the prostatic fraction of semen were processed for proteomics evaluation. LC-MS/MS analysis was performed by means of a LTQ Orbitrap Elite system. The technology combines high separation capacity and strong qualitative ability of proteins in biological samples that require deep proteome coverage. Protein classification was performed based on their functional annotations using Gene Ontology (GO). In blood plasma, semen sperm-rich fraction, and semen prostatic fraction, 59, 42 and 43 proteins, respectively, were detected. Two proteins were identified simultaneously in plasma and the semen sperm-rich fraction, 11 proteins in plasma and the semen prostatic fraction, and three proteins in the semen sperm-rich and prostatic fractions. In semen samples, most proteins were related to cell organization and biogenesis, metabolic processes or transport of ions and molecules. Most proteins were located in the cell membrane, the cytosol or the nucleus. Finally, most proteins performed functions related to binding or enzyme regulation. There were no differences between the semen sperm-rich fraction and prostatic fractions in terms of the clustering of proteins. In conclusion, a baseline reference for proteins in the semen of Beagle-breed dogs is provided. These proteins are involved mostly in supporting spermatozoan maturation, survival and motility, enhancing the reproductive performance of male animals. There appears potential for the proteomics examination of semen to become a tool in semen evaluation. This analysis may potentially identify biomarkers for reproductive disorders. This can be particularly useful in stud animals, also given its advantage as a non-invasive method.

## 1. Introduction

Reproduction is the process by which organisms produce and raise offspring, and, through it, animal species survive and flourish on Earth. Sexual reproduction includes the combination of genetic material from two parent animals, usually from two gametes. Sexual reproduction includes the fertilization of an egg cell by a sperm cell, by means of which haploid gametes fuse to produce a diploid zygote.

Semen evaluation is an integral part of the reproductive evaluation of male animals. It is performed as an adjunct test to the clinical and paraclinical (e.g., imaging examinations) examinations of the genital system of animals and aims to find out if a problem with semen or spermatozoa may be leading to a male animal’s reduced reproductive performance and to diagnose reproductive disorders in male animals.

Proteomics enables high-throughput analysis of all proteins that exist in a cell or a tissue at a particular time in a single experiment. Thus, it reveals protein expression, protein–protein interactions or post-translational modifications [1,2]. On this basis, proteins are identified during various physiological states; additionally, changes in protein presence or abundance can be recorded. Moreover, protein interaction or modification can be studied, as these result from differing normal states, response(s) of cells and tissues to changes in their microenvironment or pathological processes within a tissue.

Studies on proteomics analysis of semen have been published for boars [3,4], bucks [5], bulls [6,7], rams [8,9], stallions [10,11], and tom-cats [12].

In male dogs, early studies focused on separating seminal proteins and establishing possible associations between specific proteins and semen characteristics [13] and on the identification of the proteome of semen plasma [14]. More recently, Araujo et al. [14,15] referred to the possible effects of the various breeds of dogs on the proteomics of the spermatozoa and semen plasma, while Zmudzinska et al. [16] discussed the age-dependent variations in mixed-breed dogs.

Results of proteomics studies can provide important knowledge regarding the diversity and composition of canine seminal secretions. They will set reference values for proteins in the semen of dogs, for potential use in future research studies, as well as for diagnostic purposes, e.g., in cases of prostate neoplasia or genital infections (e.g., infection by *Brucella canis*). The results can help to clarify the pathogenesis of these disorders and to identify biomarkers for their diagnosis. Additionally, knowledge about the biochemical characteristics of seminal fluid will support a better understanding of the physiological mechanisms, by which sperm function is modulated.

The objectives of the present work were to evaluate the semen of dogs by means of proteomics methods and to compare the results with the proteomics results of blood of the animals, in order to increase available knowledge of the topic and present relevant reference values for semen samples.

## 2. Materials and Methods

### 2.1. Animals, Sample Collection, Seminological Examination

Five sexually mature, 4- to 5-year-old healthy Beagle breed dogs, with body weight ranging from 13.5 kg to 15.5 kg (median 14.2 kg) were included in the study. All dogs were routinely vaccinated against canine parvovirus infection, canine distemper, infectious hepatitis, leptospirosis and rabies, as per the recommended schedules, and treated with antiparasitics (pyrantel embonate, febantel, praziquantel (Drontal^®^ Plus) quarterly; fipronil (Frontline^®^ spot-on) monthly). Their health status was routinely assessed by means of standard clinical and laboratory examinations, i.e., complete blood counts, serum biochemical testing (total protein, albumin, blood urea nitrogen, creatinine, alkaline phosphatase, alanine transaminase, glucose), urinalysis and serological testing for leishmaniosis. The genital system of the animals was periodically examined by clinical, ultrasonographic (evaluation of echogenicity, heterogeneity, presence of hyper- or hypoechogenic foci or areas, shape distortion) and seminological examinations. In no case, abnormal findings were detected, hence animals were deemed to be healthy.

Before semen collection, a general clinical examination of the dogs was performed. A blood sample was also collected, and plasma was prepared. This was followed by a detailed clinical evaluation of the genital system, which included a detailed examination of the testes by palpation and of the prostate by digital examination. Moreover, a detailed ultrasonographic examination of the genital system was performed, by means of B-mode (evaluation of echogenicity, heterogeneity, assessment for presence of hyper- or hypoechogenic foci or areas, evaluation for shape distortion) [17] complemented with Color Doppler examination and Pulsed-Wave Doppler examination.

The ultrasonographic examination was performed with an ultrasound scanner (MyLab^®^ 30; ESAOTE SpA, Genova, Italy) fitted with a linear or a microconvex transducer for examination of the testes or the prostate, respectively. Both testes were examined with the animal in dorsal recumbency, using B-mode, Color Doppler and Pulsed-Wave Doppler mode. The hair of the scrotum or the caudal abdominal wall were clipped and hypoallergenic, acoustic coupling gel was applied. For B-mode examination, the following settings were employed: frequency 12.0 MHz (testes) or 8.0 MHz (prostate), depth 4.0 cm (testes) or 7.0 cm (prostate), overall gain 79–85% (testes) or 55–61% (prostate). Both testes and the prostate were examined in the longitudinal and transverse axes. The Color Doppler mode was then switched on and images of testes or prostate and vessels were recorded. The Pulse Repetition Frequency (PRF) was set between 1.4 to 2.8 KHz, while the frequency was automatically set at 6.6 MHz. During Pulsed-Wave Doppler examination, the same frequency was used, the PRF was adjusted appropriately, the Doppler angle was set at 40°, and the sample gate was set at 1 mm. During the examination, at least three continuous, consecutive waveforms were saved for further analysis. The following hemodynamic parameters were calculated for blood flows in the testes only: resistance index, pulsatility index, peak systolic velocity, mean velocity, end-diastolic velocity, acceleration and ratio of systolic/diastolic velocity. The blood flow was calculated automatically, after placing calipers manually at the outside vessel wall.

Semen was collected from all dogs at the end of May, by digital manipulation, using a teaser female Beagle dog for sexual stimulation [17]. The three fractions of semen of the animals: pre-sperm fraction, sperm-rich fraction, prostatic fraction, were collected separately during ejaculation, by using a 15 mL tube and plastic funnel. The pre-sperm fraction was discarded. A standard seminological evaluation was then performed in the semen samples; the following parameters were assessed: volume of the ejaculate, spermatozoal motility, total number of spermatozoa, viability of spermatozoa and presence of abnormal spermatozoa, as described in detail by Gouletsou et al. [17].

The samples from all five dogs (i.e., blood plasma, semen sperm-rich fraction, semen prostatic fraction) were then stored at −80 °C until processing for proteomic examination.

### 2.2. Sample Preparation for Proteomics Evaluation

Immediately before the start of the proteomic evaluation, all samples were thawed and then pooled for proteomic analysis. Specifically, blood plasma samples from the five dogs were pooled and one sample from that tissue was produced. The same procedure was followed for semen sperm-rich fraction samples and one sample from that tissue was produced, as well as from semen prostatic fraction. Pooling of samples (blood plasma, semen sperm-rich fraction, semen prostatic fraction) had been performed based on equal protein quantities within each sample collected from each dog.

From each of the three pooled biological samples, three technical samples were prepared for separate proteomic analyses.

### 2.3. Peptide Generation and 1-D nanoLC-MS/MS Analysis

For one-dimensional electrophoresis, each of the technical replicates of the pooled samples was analyzed separately. The amount of total soluble protein in the samples was determined by the Bradford method [18], using bovine serum albumin as standard.

The extraction of proteins and the generation of peptides were performed as described before [19,20]. In brief, the samples, at a concentration of 200 nL containing 5 μg of peptides, were treated in a water bath for 30 min, under mild sonication, with 7 M urea buffer and 80 mM triethyl ammonium bicarbonate (TEAB) [21]. The steps for the reduction and the alkylation of proteins were performed using dithiothreitol and iodoacetamide solutions, at concentrations of 10 mM and 55 mM, respectively. The final step of processing included tryptic digestion of extracted proteins for peptide generation which was performed with 5 μL of 20 μg mL^−1^ recombinant trypsin (Roche Diagnostics, Basel, Switzerland) for 16 h at room temperature.

### 2.4. LC-MS/MS Analysis

Digested samples were analyzed using a LTQ Orbitrap Elite coupled to a Dionex 3000 HPLC system (Thermo Scientific, Rockford, IL, USA). LC separation of peptides took place at a flow rate of 3 nL min^−1^ on two Thermo Scientific (Waltham, MA, USA) columns (PepMap RSLC, C18, 100 Å, 3 μm bead-packed 15 cm column and 2 μm bead-packed 50 cm column). The mobile phases A and B were, respectively, 0.1% formic acid in water and 99% acetonitrile in water. The gradient elution profile was as follows: 2.0% B (98.0% A) for 10 min, 2.0–35.0% B (98.0–65.0% A) for 325 min, 80.0% B (20.0% A) for 10 min, 2.0% B (98.0% A) for 10 min. Data were collected in the data-dependent MS/MS mode using a standard top 20 method. Full scan data were acquired at a resolving power of 60,000, with a maximum integration time of 250 ms. Scan range was fixed at 250 to 1250 *m*/*z* and peptide fragmentation was performed in a higher energy collision dissociation (HCD) mode with a normalized collision energy of 36%.

MS/MS spectra were obtained with 15,000 resolving power and a maximum integration time of 120 ms. The measurements were performed using *m*/*z* 445.120025 as lock mass. Dynamic exclusion settings were set to repeat count 1, repeat duration 30 s, exclusion duration 120 s and exclusion mass width 0.6 *m*/*z* (low) and 1.6 *m*/*z* (high).

The *.raw data files were analyzed using the Proteome Discoverer software (Thermo Scientific, Waltham, MA, USA), using the Sequest search engine, applying the *Canis lupus familiaris* *.fasta databases in the UniProt Knowledge base database (UniProtKB/SwissProt (release 2019_11) Uniprot Consortium, Cambridge, United Kingdom & Geneva, Switzerland & Washington, USA). MS/MS searches were performed using a 20 ppm parent ion mass tolerance and a 0.05 fragment mass tolerance. Trypsin was selected as the cleavage enzyme with up to 2 missed cleavage points. Cysteine methylthio modification was selected as a fixed modification and oxidation of methionine was selected as a variable. Peptide identifications were considered valid at 1% False Discovery Rate (*q*-value 0.01) (percolator maximum Delta Cn was 0.05). Values of 2.2 for doubly charged and 3.5 for triply charged peptides were used. The minimum length of acceptable identified peptides was set as 6 amino acids.

### 2.5. Protein Clustering

All proteins identified in the semen or blood samples were assigned their gene symbol via the Uniprot Knowledge dbase database [22]. Protein classification was performed based on their functional annotations using Gene Ontology (GO) for molecular function, biological process and subcellular localization. Analyses were performed for all identified seminal proteins; when more than one assignment was available, all the functional annotations were considered in the results. Gene Ontology analysis was performed as described by Anagnostopoulos et al. [19].

### 2.6. Data Management and Analysis

Data were entered into Microsoft Excel (Microsoft Corporation, Redmond, WA, USA). Basic descriptive analysis was performed. Only proteins detected in all three technical samples assessed from each tissue, were taken into account in the analysis. The number of proteins assigned per category during protein clustering was compared between the sperm-rich fraction and the prostatic fraction of semen by using cross-tabulation with Pearson’s chi-square test. Analyses were performed using SPSS v. 21 (IBM Analytics, Armonk, NY, USA). Statistical significance was defined at *p* < 0.05.

## 3. Results

### 3.1. Clinical and Seminological Findings

The examinations did not reveal any abnormal findings in the study animals. No abnormalities were detected during the clinical examination of the animals. On palpation, the testes were firm, with no palpable abnormalities therein. On digital manipulation, no abnormal structures were detected in the prostate of any animal.

**Figure 1:**
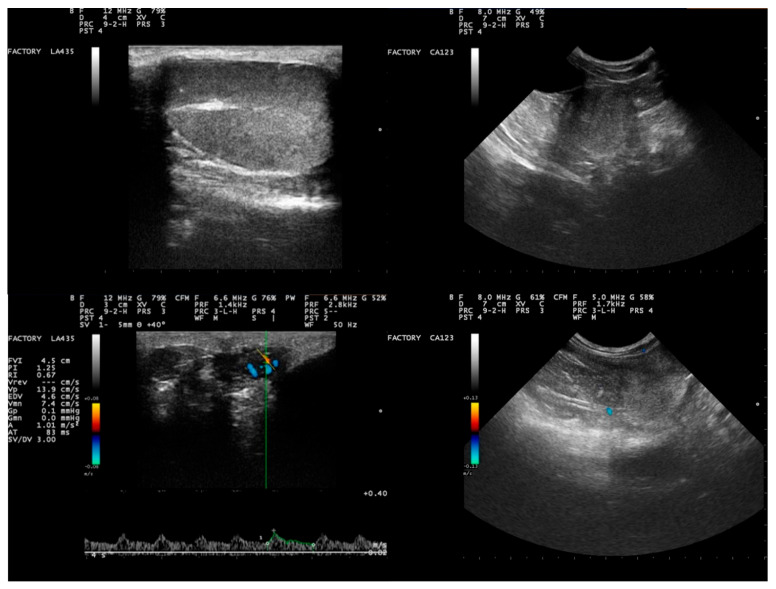
Results of ultrasonographic examination of the genital system of Beagle-breed dogs (clockwise from top left): B-mode ultrasonographic image of testicular parenchyma (sagittal section; the testicular capsule and skin were evident as a distinct hyperechogenic line rounding the parenchyma, which appears homogeneous with a central hyperechogenic line representing the mediastinum testis), B-mode ultrasonographic image of prostate parenchyma (transverse section; the gland is imaged with a regular shape and homogeneous appearance, generally hypoechoic compared to adjacent fat), Color Doppler of the prostate (longitudinal section; a few separate branches of capsular vessels are obvious in the peripheral zone of the gland), Pulsed Wave Doppler image of the looping part of the supratesticular artery; hemodynamic parameters calculated by the equipment software as seen in the image, where a low resistive flow pattern can be observed with obvious peak systolic velocity).

Ultrasonographically, no abnormal findings, either in the testicular parenchyma or in the adjacent structures or the prostate, were evident in the genital system of any dog (Figure 1). During Doppler-mode assessment, vascularization and blood flow in the testes appeared normal (Figure 1). The blood flow profile of all dogs appeared normal (Table S1).

Semen was successfully collected from all of the dogs; their libido was considered to be similar to that recorded on other occasions of semen collection by the same animals. The results of semen evaluation tests indicated that all parameters were within normal values (Table 1).

### 3.2. Proteomics Findings

In blood plasma samples, 59 proteins were detected. Furthermore, 42 proteins were detected in the semen sperm-rich fraction samples (of these, four proteins could not be fully characterized) and 43 proteins were identified in the semen prostatic fraction samples. Detailed results are in Tables S2–S4.

**Figure 2:**
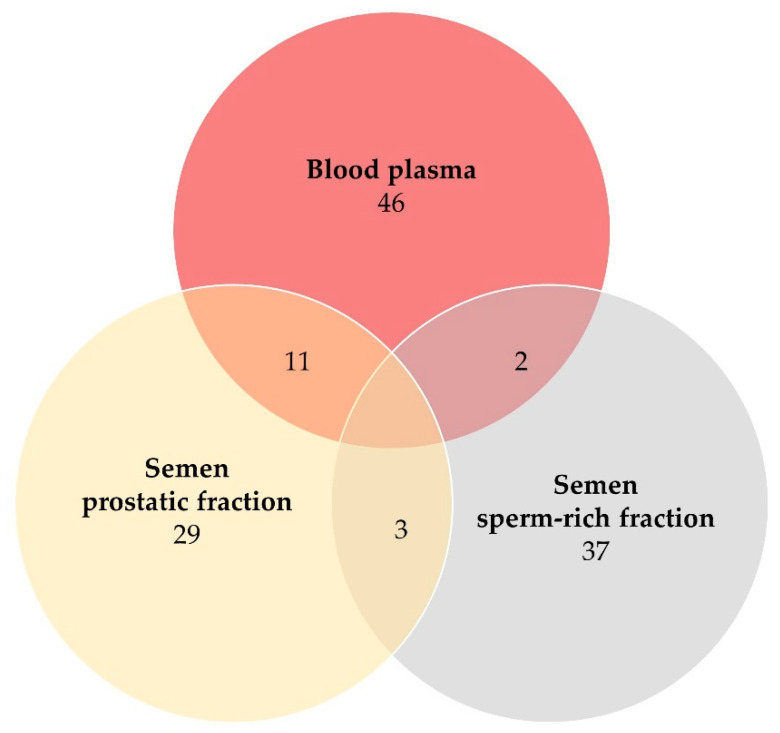
Venn diagram of numbers of proteins detected in blood plasma (dark red), semen sperm-rich fraction (grey), and semen prostatic fraction (yellow) samples from Beagle-breed dogs (protein identification by LC-MS/MS).

Two proteins were simultaneously identified in samples of blood plasma and semen sperm-rich fraction, 11 proteins in blood plasma and semen prostatic fraction and three proteins in the sperm-rich fraction and the prostatic fraction of semen samples (Table 2, Figure 2). Moreover, 37 proteins were identified exclusively in samples of sperm-rich fraction and 29 proteins were identified exclusively in samples of prostatic fraction (Table S5).

### 3.3. Clustering of Proteins

**Figure 3:**
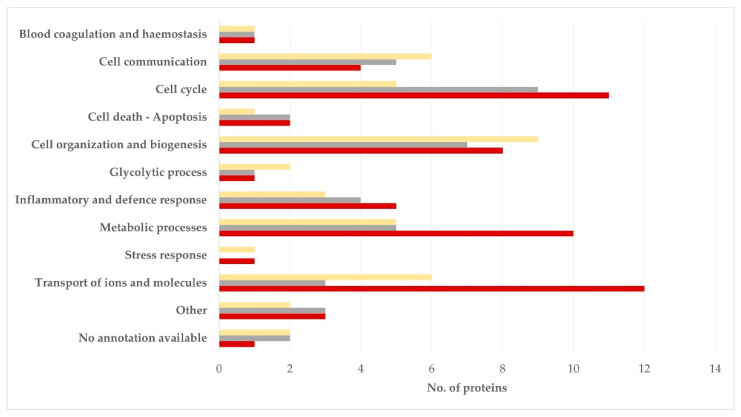
Biological processes in which proteins detected in blood plasma (dark red), semen sperm-rich fraction (grey), and semen prostatic fraction (yellow) from dogs were involved (protein identification by LC-MS/MS).

In relation to biological processes, in the semen sperm-rich fraction, most proteins were related to cell cycle (21.4%) or cell organization and biogenesis (16.7%). In the semen prostatic fraction, most proteins were related to cell organization and biogenesis (20.9%), cell communication (14.0%) or transport of ions and molecules (14.0%) (*p* = 0.92 for the difference in the frequency of the various biological processes between the two tissues) (Figure 3, Table S6).

**Figure 4:**
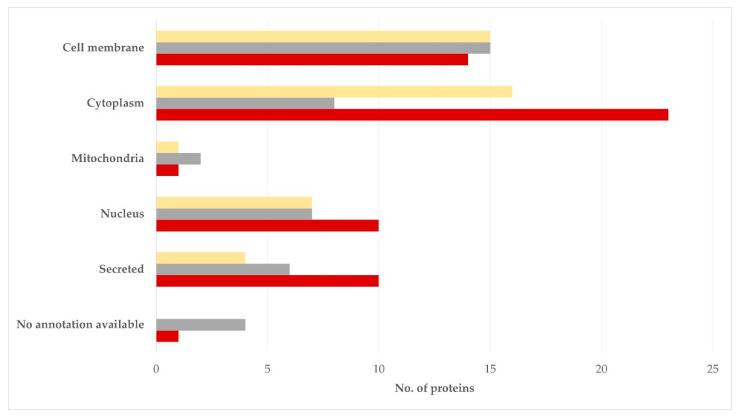
Subcellular location of proteins detected in blood plasma (dark red), semen sperm-rich fraction (grey), and semen prostatic fraction (yellow) from dogs were involved (protein identification by LC-MS/MS).

With regard to the subcellular location of the proteins identified, these were located mostly in the cell membrane, the cytosol or the nucleus: 15 (35.7%), 8 (19.0%), 7 (16.7%), respectively, for the semen sperm-rich fraction and 15 (34.9%), 16 (37.2%), 7 (16.3%), respectively, for the semen prostatic fraction (*p* = 0.54 for the difference in the frequency in the various subcellular locations between the two tissues) (Figure 4, Table S7).

**Figure 5:**
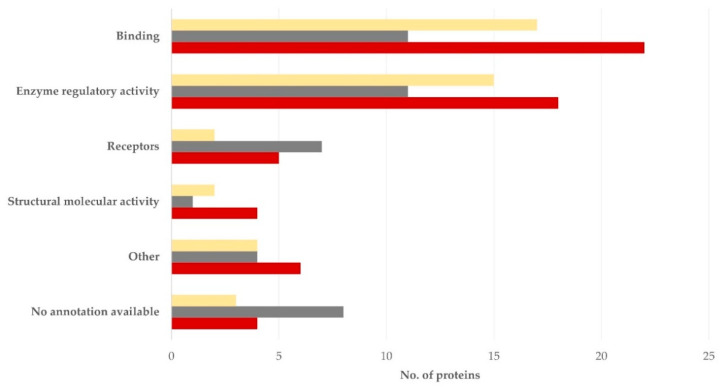
Molecular function of proteins detected in blood plasma (dark red), semen sperm-rich fraction (grey), and semen prostatic fraction (yellow) from dogs were involved (protein identification by LC-MS/MS).

Regarding the molecular function, most proteins performed functions related to binding or enzyme regulation: 11 (26.2%) for each of the two functions for the semen sperm-rich fraction and 17 (39.5%) and 11 (25.6%), respectively, for the semen prostatic fraction (*p* = 0.34 for the difference in the frequency in the various subcellular locations between the two tissues) (Figure 5, Table S8).

Details of the biological processes, the subcellular location, and the molecular function of all proteins (*n* = 128) identified in the study are presented in Table S9.

Finally, there was a tendency for more proteins with no available annotation among those detected in the semen sperm-rich fraction than among those detected in the semen prostatic fraction regarding the subcellular location (*p* = 0.038) and the molecular function (*p* = 0.095).

## 4. Discussion

### 4.1. Preamble

Τhe results of this work provide reference data for the semen of healthy dogs. The examination of the genital system of dogs is an integral part of the veterinary care provided to these animals. Semen examination is important for male dogs, particularly so for stud animals. Moreover, Beagle-breed dogs are often used in experimental research work; thus, it is worth having reference values for future use by other researchers; the use of this breed in the present study provides wider usefulness of the results. There has been a growing interest in unraveling the framework of various animal proteomes, and, among these, the semen of dogs has been described as being a biological fluid of interest from the viewpoint of proteomics analysis [23]. Hence, there is scope for implementing state-of-the-art techniques in the evaluation of the semen of such animals. In this respect, and additionally to the standard clinical and seminological examinations performed, we employed advanced imaging techniques to confirm the health of the experimental animals.

The recent increases in the availability of fractionation and identification techniques can contribute to allowing scientists to fully investigate these tissues. However, one should also consider that the lack of fully sequenced genomes in dogs can be a limiting factor in the usage of proteomic technologies. Hence, searches of nucleotides or peptide sequences in tissue samples might have failed to provide significant hits. Thus, this might be an explanation for the four uncharacterized proteins.

Nowadays, advances in proteomic technologies with modern liquid chromatography (LC)–MS/MS instruments, having undergone advancements in mass resolution, mass accuracy, fragmentation technology and speed, enable us to combine high separation capacity and strong qualitative ability of proteins in biological samples that require deep proteome coverage [21]. For the identification of proteins, LC-MS/MS analysis by means of a LTQ Orbitrap Elite equipment was employed. The increased accuracy of the technique indicated the detection of the entirety of proteins: 82 proteins in total in the semen, specifically, 43 in the prostatic fraction and 42 in the sperm-rich fraction. Then, existing gene ontology information was used to constellate proteins identified in the semen samples in accord with their biological process, subcellular location and molecular function. Gene ontology classification of the unique genes in each constructed semen proteome database revealed their scientific meaning and provided information on characteristics of the protein ingredients of semen.

It is noteworthy that, previously, other authors have reported a higher number of proteins [15,24] than the number of proteins identified in the present study. Those authors have performed their studies in other breeds of dogs, specifically Rottweiler, German-Shepherd [24], Golden Retriever, Great Dane, Bernese Mountain and Maremmano-Abruzzese SheepDog [15] (i.e., large or giant dogs), but with a disagreement between the two works in the proteins detected in semen of respective breeds. Araujo et al. [15] also stated that not only differences in proteins would be evident between breeds, but also between individuals of the same breed, due to heterogeneity in purebred animals. Hence, the lack of standardization of proteomics technologies with various sample preparation protocols (e.g., protein extraction) and identification methods can lead to a variety of results. These can justify the differences observed in results between the present study and those of previous ones. Moreover, pre-analytical variables, which include all steps of the procedures carried out before the analysis of the biofluid, e.g., sample collection, handling and storage, may influence the outcomes of proteomics work [25]. In the present study, semen collection, handling and storage were applied as previously described [17], by following accredited procedures, consistently employed at the Department of Obstetrics and Reproduction of the Faculty.

### 4.2. Significance of Proteins Detected in Semen Samples

As expected, the proteins detected were involved in various functions promoting the reproductive activity of male animals. In the sperm-rich fraction, proteins related to the structure of sperm or their motility were identified; in some cases, the same proteins had been detected in previous studies from samples obtained from the genital system of males, in dogs or other mammals. Various membrane proteins among those detected had been individually found to be localized in the plasma membrane of spermatozoa. Such proteins have important functions, for example serving as receptors, ion channels, structural proteins and enzymes [26,27]. In the prostatic fraction, proteins are related to protection of sperms from adverse stimuli or promote their activity within the genital system of female animals. Details of some of the proteins identified are discussed below.

Among the proteins that were detected in both the sperm-rich and the prostatic fractions, apolipoprotein E can play various roles in the genital system (e.g., lipid transport, participation in enzymic reactions as a co-factor), although these have not been fully elucidated; the protein was detected in the testes, the epididymides (epithelial cells and interstitium), the seminal vesicles, the prostate of men [28], as well as in sperm-rich and prostatic fractions of dogs (Rottweiler, German-Shepherd) [24]. There are few data available for the potential role of keratin, type II cytoskeletal 1 in the genital system; in humans, higher expression levels of the protein were associated with reduced motility of spermatozoa [29]; keratin type II cytoskeletal 5, a cell structure protein, was detected in the sperm flagellum [30,31] and considered to contribute to its dense fibre composition [32]; the protein was also detected repeatedly in the seminal plasma and the spermatozoa of dogs (Rottweiler, German-Shepherd, Golden Retriever, Great Dane, Bernese Mountain, Maremmano-Abruzzese SheepDog) [14,15,24]. Nitric oxide synthase is involved in the spermatogenesis and the apoptosis of Sertoli and germ cells [33] and plays a role in the regulation of germ cell numbers and testicular size [34], through the oxidation of l-arginine by nitric oxide synthases [35].

The seminal plasma includes proteins necessary for the function and the survival of the spermatozoa [36,37]. In this respect, they participate in a variety of functions: binding onto the sperm surface after ejaculation and participating in sperm capacitation, acrosome reaction and sperm-ovum fusion [38,39].

Among the proteins that were detected in the sperm-rich fraction, aggrecan core protein and cadherin-1 are calcium binding proteins, which is a similar finding to that of another study in dog semen (Rottweiler, German-Shepherd) [16,24]. Sperm maturation is regulated by Ca^2+^-signaling pathways [40,41], in which Ca^2+^ ions bind to Ca^2+^-binding proteins. These refer to the regulation of flagellar beating and the acrosome reaction of sperms [42,43]; for example, cadherin is responsible for cell adhesion, as well as participating in germline development and gametogenesis and fertilization [44]. Another calcium binding protein, acrosin-binding protein, has been previously detected in the semen of dogs (Bernese Mountain) [15].

The presence of cytosol in spermatozoa [45], specifically on their surface [46], is possibly associated with the induction of the acrosome reaction. The aminopeptidases have been reported to be potentially related to adverse effects caused to spermatozoa by increased temperatures [47]. While cytosol aminopeptidase has been found to be expressed during dry conditions, the expression of lysine-specific demethylase 5D in the membrane of spermatozoa was more intense during rainy periods [48].

Cytospin A may participate in the stabilization of microtubules and the organization of actin cytoskeleton. It is involved in sperm migration and participates in cytokinesis and the organization of the spindle.

Nephrocystin-1 (Fragment) is involved in spermatogenesis; in particular, it is required for the differentiation of early elongating spermatids into spermatozoa [49]. Moreover, the protein PALS1, which interacts with nephrocystin, regulates flagellated sperm motility [50].

Tektin-2 is a structural component of sperm, specifically part of axonemal proteins [51]. The protein participates in the assembly or attachment of the inner dynein arm complex to microtubules in sperm flagellar motility [52].

Ubiquitin is secreted in association with the epididymosomes [53]. Isoforms of the protein were previously detected in sperm-rich and prostatic fractions in dogs (Rottweiler, German Shepherd) [24] and in the spermatozoa head, playing a role in the acrosome reaction and gamete binging, specifically participating in the prevention of polyspermy in men [54]. Protein ubiquitination has been reported in the membrane [55] and other regions [56] of spermatozoa; it has been suggested that the process plays a role in the normal function of spermatozoa [57,58].

Abnormal spindle-like microcephaly associated protein homolog (ASPM) is a protein expressed in a variety of embryonic and adult tissues, including canine epididymal fluid [16], and is upregulated in cases of neoplastic disorders [59]. Lack of a functional ASPM may affect the fidelity of chromosome segregation, leading to reduced ability of fetal stem cells to produce neurons [59]. ASPM also plays a role in sperm flagellar function [60].

The prostatic fluid includes proteins secreted from the prostate. These promote sperm activation and function subsequently to the ejaculation, i.e., in the genital tract of females [61].

Among the proteins that were detected in the prostatic fraction, actin cytoplasmic 1 is a protein related to cell organization [16,24,62]. It participates in various cellular processes, e.g., establishment and maintenance of cell junctions and cell shape, cell division and cytokinesis, cell motility and muscle contraction. The presence of actin in the tail of spermatozoa might be important for the regulation of sperm motility, and its presence in the head suggests a possible involvement in the acrosome reaction. The polymerization of actin is important for the initiation of the motility of sperms during their maturation in the epididymis [63]. Finally, actin is associated with membrane structures [64] and is also involved in intracellular changes associated with the capacitation of spermatozoa and the acrosome reaction [65]. In previous studies, cytoskeletal proteins, e.g., proteins of the tubulin family, were detected (tubulin alpha-3E chain in Maremmano-Abruzzese SheepDog, tubulin alpha-3 chain in Bernese Mountain) [15]; these are the main structural components of microtubules and are involved with the flagella movement.

Canine arginine esterase, a kallikrein detected in the prostatic fraction, found to be the protein in the higher abundance in canine seminal plasma [24], is an immunological marker for assessing the normal function of the prostate gland [66]; it is similar to the prostatic specific antigen, which in men is an important marker for prostate cancer [67]. Moreover, the protein has a homology with heparin-binding proteins (SSPs-7) detected in the semen of stallions [68]; canine esterase has the ability to bind with zinc ions [67], thus contributing to the normal function of the genital system. Zinc plays an important role in the normal function of the genital system of male animals, specifically in the development of testes, the spermatogenesis and the motility of spermatozoa, through stabilizing the cell membrane and the nuclear chromatic of spermatozoa [69,70]. Another protein, zinc transporter SLC39A7, detected in the prostatic fraction, is also involved in zinc metabolism [71].

Cathepsin L1, originating from the accessory sex glands [24,72], interacts with the spermatozoa. It possibly facilitates penetration of the barriers set by the cumulus cells and the zona pellucida [73].

Several isoforms of clusterin have been detected in the seminal plasma of dogs [14,15,16,24] and bulls [6,74,75] and participate in the protection of spermatozoa through secretion in response to cellular damage and heat-shock [76]. Clusterin has been detected in samples from crossbreed dogs [16]. The protein constitutes the bulk of protein expression activity of the epididymis [77]. It plays a role in shielding cells against protein precipitation and attacks by the immune system [78], as well as supporting the removal of defective spermatozoa in the epididymis [79].

Coiled-coil domain-containing protein has a regulatory role for assembling of the dynein regulatory complex and inner dynein arm complexes, which play a role in the motility of spermatozoa [80].

Heat shock protein 70 kDa has been reported as a component of spermatozoa in various species, including dogs [24], specifically in the acrosome [81,82] and participates in gamete interaction and fertilization [83]. The protein plays a role in maintaining the folding stage for mitochondrial proteins and is involved in oxidation and/or reduction activities in association with energy metabolism [84].

Serum paraoxonase/arylesterase 2 is a protein carried on HDL (high-density lipoprotein) cholesterol and believed to be a source of the antioxidant characteristics of that molecule [85]. It has been detected with a higher expression in males with normal semen characteristics [85,86].

Tight junction ZO_3_ is one of the scaffolding proteins, linking tight junction transmembrane proteins (e.g., claudins), junctional adhesion molecules and occludin to the actin cytoskeleton [87]. Tight junctions are part of the blood–epididymis barrier, which mediates the paracellular transport of ions and solutes and controls the differentiation of epithelial cells, that way contributing to the establishment of the environment of the lumen.

Obviously, the proteins detected fulfilled functions and roles taking place in healthy animals. The identification of proteins exclusively in the sperm-rich fraction or in the prostatic fraction reflects the different functions played by the proteins in each of these tissues, as detailed above for some of them, e.g., cadherin-1, cytospin-A and tektin-1 (among the proteins found in the sperm-rich fraction) and aminopeptidase N and procathepsin L (among the proteins found in the prostatic fraction). The different physiological role of each of these two tissues is compatible with the increased number of proteins involved in the cell cycle for the sperm-rich fraction and in the transport of ions and molecules in the prostatic fraction (Table S6).

Semen is a particularly complex secretion, and includes various molecules from the male genital system. Apart from the spermatozoa, semen also includes secretions from the seminiferous tubule lumen/epididymis/*ductus deferens*, as well as from the accessory glands of the male genital system (ampullary glands, bulbourethral glands, prostate, seminal vesicles, urethral glands) [88]. These secretions participate in the regulation of various mechanisms, which may take place within the male genital system, for example during the maturation of spermatozoa in the epididymis [89], or after ejaculation, for example for the protection of spermatozoa during their passage through the female genital tract or for capacitation of spermatozoa [90,91]. Thus, problems in protein expression would result in dysfunction of the genital system of male dogs. Consequently, proteins in semen may be used as potential biomarkers for cases of reproductive disorders in male animals (e.g., functional subfertility, reproductive infections).

## 5. Conclusions

A baseline reference for proteins in the semen of Beagle-breed dogs was provided. These proteins are mostly involved in supporting spermatozoan maturation, survival and motility, enhancing the reproductive performance of male animals. Future work can focus on the quantification of proteins identified in semen, for example, with Western Blot analysis and will compare findings with results in samples from animals with suboptimal fertility. This provides potential for the use of proteomics examination of semen as a tool in semen evaluation. This could be particularly useful in stud animals, also given its advantage as a non-invasive method.

## Figures and Tables

**Table 1:** Results of evaluation of semen samples from dogs into the study.

Parameter	Median (Min.–Max.)
Volume of the sperm-rich fraction (mL)	1.80 (0.90–2.10)
Spermatozoal motility (%)	87.0 (80.0–95.0)
Total number of spermatozoa (mL^−1^)	363,000 (210,000–490,000)
Proportion of dead spermatozoa (%)	6.7 (5.0–11.0)
Proportion of abnormal spermatozoa (%)	9.0 (8.0–12.0)

**Table 2:** Proteins simultaneously identified in two tissues among those assessed: blood plasma (BP), semen sperm-rich fraction (SF), and semen prostatic fraction (PF) from Beagle-breed dogs (protein identification by LC-MS/MS).

Accession No.	Description	Simultaneous Detection of Proteins		
		BP	SF	PF
P62286	Abnormal spindle-like microcephaly associated protein homologue	Yes	Yes	No
O18840	Actin, cytoplasmic 1	Yes	No	Yes
P18649	Apolipoprotein E	No	Yes	Yes
P09582	Arginine esterase	Yes	No	Yes
P25473	Clusterin	Yes	No	Yes
E2R1I5	Coiled-coil domain-containing protein 39	Yes	No	Yes
Q8WN22	DNA-dependent protein kinase catalytic subunit	Yes	No	Yes
Q28259	Glyceraldehyde-3-phosphate dehydrogenase	Yes	No	Yes
Q659K0	G2/mitotic-specific cyclin-B3	Yes	No	Yes
Q7YQC6	Heat shock 70 kDa protein 1	Yes	No	Yes
Q6EIY9	Keratin, type II cytoskeletal 1	No	Yes	Yes
Q9TU19	Nephrocystin-1(Fragment)	Yes	Yes	No
O62699	Nitric oxide synthase, inducible	No	Yes	Yes
P54832	Serum paraoxonase/arylesterase 2	Yes	No	Yes
O62683	Tight junction protein ZO-3	Yes	No	Yes
P63050	Ubiquitin-60S ribosomal protein L40	Yes	No	Yes

Yes: protein identified in samples from respective tissue, No: protein not identified in samples from respective tissue.

## Data Availability

All data are presented in Supplementary Materials.

## Supplementary Materials

The following supporting information can be downloaded at: https://www.mdpi.com/article/10.3390/vetsci9120697/s1, Table S1: Results of haematological parameters in Doppler mode examination of dogs into the study; Table S2: Proteins (*n* = 59) identified in blood plasma from male dogs; Table S3: Proteins (*n* = 43) identified in prostatic fraction of semen from male dogs; Table S4: Proteins (*n* = 42) detected in semen-rich fraction of semen from male dogs; Table S5: Proteins identified exclusively in semen sperm-rich fraction or in semen prostatic fraction from male dogs. Table S6: Biological processes in which proteins detected in blood plasma, semen prostatic fraction and semen sperm-rich fraction from male dogs, were involved; Table S7: Subcellular location of proteins detected in blood plasma, semen prostatic fraction and semen sperm-rich fraction from male dogs; Table S8: Molecular function of proteins detected in blood plasma, semen prostatic fraction and semen sperm-rich fraction from male dogs; Table S9: Details of the biological process, the subcellular location and the molecular function of all proteins(*n* = 128) identified in the study.
