# Supplementary material for: Proteomics Evaluation of Semen of Clinically Healthy Beagle-Breed Dogs
Source: Vet Sci. 2022 Dec 15;9(12):697. doi: 10.3390/vetsci9120697 (PMC9785154; doi:10.3390/vetsci9120697)
Supplement: Supplementary file 1 [file vetsci-09-00697-s001.zip › vetsci-2059780-supplementary.pdf]

veterinary sciences

MDPI

Proteomics Evaluation of Semen of Clinically Healthy Beagle-Breed Dogs

Pagona G. Gouletsou [1,†], George Th. Tsangaris [2,†], Eleni I. Katsarou [1], Maria V. Bourganou [3], Mariana S. Barbagianni [1], Athina P. Venianaki [1], Efterpi Bouroutzika [2], Athanasios K. Anagnostopoulos [2], George C. Fthenakis [1] and Angeliki I. Katsafadou [3,*]

Table S1. Results of blood flow profile parameters in Doppler mode examination of dogs into the study.

Parameter	Median (min. – max.)
Resistance index	0.61 (0.53 – 0.67)
Pulsatility index	1.02 (0.70 – 1.25)
Peak systolic velocity (cm s^{-1})	13.2 (12.4 – 14.1)
Mean velocity (cm s^{-1})	6.9 (6.0 – 7.9)
End diastolic velocity (cm s^{-1})	5.3 (4.2 – 6.8)
Acceleration (m s^{-2})	1.06 (0.95 – 1.17)
Ratio of systolic / diastolic velocity	2.65 (1.76 – 3.20)
Blood flow (mL min^{-1})	9 (4 – 12)

Table S2. Proteins (n = 59) identified in blood plasma from male dogs (protein identification by LC-MS/MS) (in alphabetical order).

Accession No.	Description name	Gene Name	Coverage	Proteins	Unique Peptides	Peptides	PSMs [1]	Area	AAs [2]	MW [3] [kDa]	calc. pI [4]
Q5TJF5	(3R)-3-hydroxyacyl-CoA dehydrogenase	HSD17B8	7.72	1	1	1	1	1.623E7 [5]	259	26.7	7.25
E2QXF3	60S ribosomal protein L15	RPL15	8.82	1	1	1	2	2.073E8	204	24.1	11.62
P62286	Abnormal spindle-like microcephaly-associated protein homolog	ASPM	1.96	1	4	4	25	1.489E9	3469	407.3	10.61
Q8HY67	Acidic leucine-rich nuclear phosphoprotein 32 family member A	ANP32A	4.42	1	1	1	1	2.148E8	249	28.5	4.11
O18840	Actin, cytoplasmic 1	ACTB	36.27	1	11	11	11	1.521E8	375	41.7	5.48
Q29471	Annexin A13	ALB	7.07	1	8	8	312	2.752E9	608	68.6	5.69
P02648	Apolipoprotein A-I	ANXA13	3.48	1	1	1	5	4.267E8	316	35.5	5.67
P09582	Arginine esterase	APOA1	14.29	1	3	3	42	2.963E8	266	30.2	5.30
Q32KH9	Arylsulfatase G	*N/A*	13.46	1	5	5	382	5.297E8	260	28.7	7.78
A1E2V0	Baculoviral IAP repeat-containing protein 3	ARSG	2.99	1	1	1	3		535	58.2	6.74
Q5TJE8	Beta-1,3-galactosyltransferase 4	BIRC3	2.65	1	1	1	4	2.530E9	604	67.9	6.20
Q95153	Breast cancer type 1 susceptibility protein homolog	B3GALT4	2.35	1	1	1	1	1.857E7	383	42.4	8.72
E2RA18	Calmegin	BRCA1	0.59	1	1	1	1		1878	208.3	5.59
P24643	Calnexin	CLGN	2.90	1	1	1	1		620	70.9	4.67
O77746	cGMP-specific 3',5'-cyclic phosphodiesterase	CANX	1.69	1	1	1	1	4.303E8	593	67.6	4.61
P25473	Clusterin	PDE5A	3.12	1	1	1	1		865	98.2	6.06
E2R1I5	Coiled-coil domain-containing protein 39	CLU	1.80	1	1	1	1		445	51.8	5.91
Q3LSL6	C-X-C chemokine receptor type 4	CCDC39	1.06	1	1	1	53	1.004E9	939	110.3	6.61
Q8WN22	DNA-dependent protein kinase catalytic subunit	CXCR4	1.70	1	1	1	14	2.287E7	353	40.1	8.21
P41148	Endoplasmin	PRKDC	0.24	1	1	1	1	4.491E7	4144	472.8	7.23
Q9GL25	Epididymal sperm-binding protein 1	HSP90B1	9.58	1	6	6	6	4.362E7	804	92.5	4.87
P68213	Fibrinogen alpha chain	ELSPBP1	9.80	1	1	1	1	2.091E7	245	28.7	7.40
P02677	Fibrinogen beta chain (Fragment)	FGA	67.86	1	2	2	4	4.049E8	28	3.0	6.46
Q659K0	G2/mitotic-specific cyclin-B3	FGB	61.29	1	1	1	1	1.677E7	31	3.7	5.00
A2IBY7	Gamma-crystallin S	CCNB3	1.43	1	1	1	1435	44.69E9	1330	152.0	8.94
A0PA85	Glutamate decarboxylase 1	CRYGS	7.87	1	1	1	2	5.263E9	178	21.0	6.86
Q28259	Glyceraldehyde-3-phosphate dehydrogenase	GAD1	3.37	1	1	1	1	2.058E8	594	66.8	7.31
Q7YQC6	Heat shock 70 kDa protein 1	GAPDH	4.50	1	1	1	1	3.607E8	333	35.8	8.12
P60529	Hemoglobin subunit alpha	HSPA1	1.72	1	1	1	2	2.169E8	641	70.0	5.66
P60524	Hemoglobin subunit beta	HBA	11.35	1	2	2	3	9.449E6	141	15.2	8.06
P01784	Ig heavy chain V region GOM	HBB	5.48	1	1	1	1	1.473E7	146	16.0	8.05
Q95LF0	Interleukin-13 receptor subunit alpha-2	*N/A*	16.67	1	1	1	2	8.823E7	114	12.4	5.40

Q5XNR9	Leukemia inhibitory factor receptor	IL13RA2	1.81	1	1	1	2	1.573E8	386	45.1	5.14
P35521	Methylosome subunit pICln	LIFR	3.10	1	2	2	106	13.41E9	1097	123.6	5.64
P23294	Myelin proteolipid protein	CLNS1A	11.06	1	1	1	1	5.176E8	235	25.9	4.16
P49824	Myosin-7	PLP1	3.97	1	1	1	1		277	30.1	8.35
Q9TU19	Nephrocystin-1 (Fragment)	MYH7	0.88	1	1	1	1		1935	222.8	5.73
O02813	Neuropeptide Y receptor type 1	NPHP1	3.72	1	3	3	15	1.075E9	565	63.9	4.96
Q0ZAQ8	Peroxisome proliferator-activated receptor delta	NPY1R	4.19	1	1	1	1	7.980E8	382	44.2	7.77
P80009	Plasminogen (Fragment)	PPARD	1.59	1	1	1	1		441	49.7	8.05
Q6Q308	Pleckstrin	PLG	3.00	1	1	1	1	1.628E8	333	36.7	8.10
Q9GLW0	Progesterone receptor	PLEK	2.85	1	1	1	1	4.079E8	351	40.2	8.28
Q5XXR2	Pro-MCH	PGR	1.38	1	1	1	1	6.102E8	939	98.4	8.37
Q5PU49	Protein kinase C delta type	PMCH	12.73	1	1	1	1	1.393E7	165	18.6	7.96
Q29536	Pyruvate kinase PKLR	PRKCD	2.23	1	1	1	24	9.492E9	674	77.3	7.84
P24409	Ras-related protein Rab-10	PKLR	1.92	1	1	1	1	4.766E7	574	62.0	7.75
P61105	Ras-related protein Rab-2A	RAB10	11.00	6	2	2	2	1.420E7	200	22.6	8.38
P06871	Serine protease 1	RAB2A	6.60	1	1	1	1	1.339E7	212	23.5	6.54
P49822	Serum albumin	PRSS1	4.07	1	1	1	1	1.771E8	246	26.2	8.07
P54832	Serum paraoxonase/arylesterase 2	PON2	5.08	1	1	1	18	4.680E9	354	39.6	6.09
P61010	Signal recognition particle 54 kDa protein	SRP54	3.57	1	1	1	6	7.457E9	504	55.7	8.75
P50997	Sodium/potassium-transporting ATPase subunit alpha-1	ATP1A1	1.47	2	1	1	1		1021	112.6	5.86
Q5WR10	Spliceosome RNA helicase DDX39B	DDX39B	4.91	1	1	1	94	2.117E9	428	49.0	5.67
O62683	Tight junction protein ZO-3	TJP3	1.56	1	1	1	1	3.456E9	898	98.4	7.31
Q697L1	Transient receptor potential cation channel subfamily V member 1	TRPV1	1.90	1	1	1	6	3.018E9	840	95.2	7.30
E2RDV1	Tudor domain-containing protein 7	TDRD7	1.07	1	1	1	1	4.359E8	1125	126.6	7.64
P63050	Ubiquitin-60S ribosomal protein L40	UBA52	38.28	1	5	5	6	1.107E8	128	14.7	9.83
Q5TJF0	Vacuolar protein sorting-associated protein 52 homolog	VPS52	1.11	1	1	1	247	1.934E8	723	82.2	5.99
Q9XSR1	Zinc finger protein	ZNF252	2.29	1	1	1	3	11.66E9	873	99.4	8.65

1: Peptide spectrum matches

2: Amino acids

3: Molecular weight

4: calculated isoeletric point

5: E*n* is equivalent to 10^n.

Table S3. Proteins (*n* = 42) identified in sperm-rich fraction of semen from male dogs (protein identification by LC-MS/MS) (in alphabetical order).

Accession No.	Description name of fully characterized proteins	Gene Name	Coverage	Pro-teins	Unique Peptides	Pep-tides	PSMs [1]	Area	AAs [2]	MW [3] [kDa]	calc. pI [4]
E2RKA8	60S ribosomal protein L32	RPL32	5.19	1	1	1	48	7.532E8 [5]	135	15.8	11.33
P62286	Abnormal spindle-like microcephaly-associated protein homolog	ASPM	0.23	1	1	1	1	9.734E6	3469	407.3	10.61
Q6W3F4	Adenosine receptor A2b	ADORA2B	8.43	1	1	1	7	3.624E8	332	36.4	8.29
Q28343	Aggrecan core protein	ACAN	0.69	1	1	1	24	2.202E7	2333	240.4	4.26
Q5F2N4	Alpha-(1,3)-fucosyltransferase 10	FUT10	2.09	1	1	1	1	4.179E6	478	56.0	8.37
P58027	Amine oxidase [flavin-containing] A	MAOA	3.61	1	1	1	3	7.734E7	527	60.0	8.02
P18649	Apolipoprotein E	APOE	3.10	1	1	1	1	1.123E7	323	37.2	5.30
P61797	Aprataxin	APTX	5.26	1	1	1	67	3.106E8	342	39.0	8.82
Q45T69	Bcl-2-like protein 2	BCL2L2	10.36	1	1	1	3	8.300E7	193	20.8	5.58
Q5TJG6	Bromodomain-containing protein 2	BRD2	4.36	1	1	1	1		803	88.2	9.06
F1PAA9	Cadherin-1	CDH1	1.92	1	1	1	8	3.801E5	885	97.7	4.81
Q5ECR9	C-C chemokine receptor type 5	CCR5	1.70	1	1	1	1	3.874E6	352	40.4	9.32
O18806	Coagulation factor VIII	F8	2.22	1	2	2	2		2343	265.7	6.77
Q5U820	Cystic fibrosis transmembrane conductance regulator	CFTR	1.15	1	1	1	2	1.231E8	1483	168.4	8.90
Q2KNA0	Cytospin-A	SPECC1L	0.72	1	1	1	3	1.656E7	1117	124.6	5.76
Q767J3	Deoxyribonuclease-1	DNASE1	10.21	1	1	1	2	6.938E7	284	31.5	5.45
P13206	Endothelin-1	EDN1	4.95	1	1	1	1	1.954E7	202	23.0	9.54
Q64HP0	G1/S-specific cyclin-D1	CCND1	4.75	1	1	1	1		295	33.3	5.44
P30552	Gastrin/cholecystokinin type B receptor	CCKBR	6.84	1	1	1	1	6.379E5	453	48.5	9.85
Q4PRC2	Glutamate decarboxylase 2	GAD2	2.05	1	1	1	1	1.015E8	585	65.4	6.79
P17124	Histamine H2 receptor	HRH2	11.42	1	1	1	1	1.507E7	359	40.2	9.09
Q29467	Inositol polyphosphate-5-phosphatase A	INPP5A	2.18	1	1	1	2	7.024E7	412	47.7	7.36
Q28268	Interleukin-12 subunit beta	IL12B	2.13	1	1	1	1		329	37.4	7.17
Q6EIY9	Keratin, type II cytoskeletal 1	KRT1	3.55	1	1	1	1	1.312E8	619	63.8	7.84
Q1ZZS1	Lengsin	LGSN	4.88	1	1	1	12		574	63.9	6.09
Q30DN6	Lysine-specific demethylase 5D	KDM5D	1.75	1	1	1	1	8.653E6	1545	175.5	5.74
Q9TU19	Nephrocystin-1 (Fragment)	NPHP1	6.37	1	1	1	1	4.159E7	565	63.9	4.96
O62699	Nitric oxide synthase, inducible	NOS2	0.69	1	1	1	1	8.335E6	1154	131.8	8.10
F1P7W1	Olfactory receptor	OR13C11B	10.97	1	1	1	1		319	35.4	8.65
Q6Q7P4	Protein 4.1	EPB41	3.95	1	1	1	325	3.624E8	810	90.6	5.55
E2QY99	Protein PALS1	PALS1	1.33	1	1	1	3	6.161E7	675	77.2	6.18
Q5XM32	Relaxin receptor 2	RXFP2	4.88	1	2	2	5	3.861E7	737	84.1	8.38
Q75W64	Single-stranded DNA cytosine deaminase	AICDA	7.58	1	1	1	1		198	23.9	9.35

P23685	Sodium/calcium exchanger 1	SLC8A1	2.89	1	1	1	18	7.085E7	970	107.9	4.96
Q9TTW3	Tektin-1	TEKT1	2.82	1	1	1	3	2.136E7	426	49.0	6.16
A8C750	Thyroid adenoma-associated protein homolog	THADA	1.49	1	1	1	40	3.624E8	1948	218.8	6.46
Q8HYB7	Thyroid peroxidase	TPO	3.60	1	1	1	1		944	101.3	7.81
Q5I2M8	Toll-like receptor 9	TLR9	3.78	1	1	1	1		1032	115.2	9.03

**Accession No.**	**Description name of proteins not fully characterized**	**Gene Name**	**Coverage**	**Pro-teins**	**Unique Peptides**	**Pep-tides**	**PSMs**	**Area**	**AAs**	**MW [kDa]**	**calc. pI**
F1PA44	Coiled-coil domain containing protein 112 (Submitted name)	CCDC112	2.12	1	1	1	1		425	50.8	9.72
F1P660	Mediator complex subunit 23 (Submitted name)	MED23	3.14	1	1	1	1		1371	156.7	7.31
F1PH13	Potassium channel tetramerization domain containing 8 (Submitted name)	KCTD8	1.89	1	1	1	1		475	51.9	8.51
F1PAB2	Transport and Golgi organization 6 homolog (Submitted name)	TANGO6	1.10	1	1	1	1		1091	120.7	6.14

1: Peptide spectrum matches

2: Amino acids

3: Molecular weight

4: calculated isoeletric point

5: E*n* is equivalent to 10^n.

Table S4. Proteins (n = 43) identified in prostatic fraction of semen from male dogs (protein identification by LC-MS/MS) (in alphabetical order).

Accession No.	Description name	Gene Name	Coverage	Pro-teins	Unique Peptides	Pep-tides	PSMs [1]	Area	AAs [2]	MW [3] [kDa]	calc. pI [4]
Q6XXX9	5-hydroxytryptamine receptor 1A	HTR1A	7.09	1	1	1	1	3.207E8 [5]	423	46.3	9.14
Q5TJE9	40S ribosomal protein S18	RPS18	7.89	1	1	1	2	8.889E6	152	17.7	10.99
D0VWQ5	60S ribosomal protein L19	RPL19	4.59	1	1	1	1	1.948E7	196	23.5	11.47
O18840	Actin, cytoplasmic 1	ACTB	17.33	1	4	4	5	1.408E8	375	41.7	5.48
P79143	Aminopeptidase N	ANPEP	1.44	1	1	1	1		975	110.2	6.18
Q7YRF1	AP-3 complex subunit beta-1	AP3B1	1.65	1	1	1	1	9.415E6	1091	121.1	5.76
E2RE76	Apolipoprotein A-IV	APOA4	3.44	1	1	1	1		378	42.5	6.09
P18649	Apolipoprotein E	APOE	5.57	1	1	1	1	9.748E7	305	35.3	5.24
P09582	Arginine esterase	N/A	28.08	1	15	15	301	107.1E9	260	28.7	7.78
P25473	Clusterin	CLU	3.82	1	1	1	1	1.718E7	445	51.8	5.91
E2R1I5	Coiled-coil domain-containing protein 39	CCDC39	1.06	1	1	1	3	1.047E9	939	110.3	6.61
P49819	Dihydrolipoyl dehydrogenase, mitochondrial	DLD	2.55	1	1	1	1	5.787E6	509	54.1	7.84
Q8WN22	DNA-dependent protein kinase catalytic subunit	PRKDC	0.60	1	1	1	1		4144	472.8	7.23
E2RQ08	Dolichyl-diphosphooligosaccharide--protein glycosyltransferase subunit 1	RPN1	2.97	1	1	1	1	1.227E8	607	68.5	6.42
Q4VSN4	Dual serine/threonine and tyrosine protein kinase	DSTYK	1.83	1	1	1	1		931	104.9	6.71
Q659K0	G2/mitotic-specific cyclin-B3	CCNB3	1.43	1	1	1	1		1330	152.0	8.94
Q28259	Glyceraldehyde-3-phosphate dehydrogenase	GAPDH	4.50	1	1	1	1	1.826E8	333	35.8	8.12
P62872	Guanine nucleotide-binding protein G(I)/G(S)/G(T) subunit beta-1	GNB1	8.24	1	1	1	1	1.387E8	340	37.4	6.00
Q7YQC6	Heat shock 70 kDa protein 1	HSPA1	1.72	1	1	1	1	1.401E8	641	70.0	5.66

Q28270	Hematopoietic progenitor cell antigen	CD34	3.86	1	1	1	1	4.782E7	389	41.3	5.88
P39881	Homeobox protein cut-like 1 (Fragment)	CUX1	1.85	1	1	1	1	7.528E7	975	105.4	9.89
Q00M95	Inactive rhomboid protein 2	RHBDF2	2.18	1	1	1	1	3.314E9	827	93.5	8.76
Q6EIY9	Keratin, type II cytoskeletal 1	KRT1	3.55	1	1	1	1	1.312E8	619	63.8	7.84
Q076A6	Myosin-1	MYH1	1.65	2	2	2	2		1939	223.0	5.76
Q258K2	Myosin-9	MYH9	0.77	1	1	1	1		1960	226.3	5.60
O62699	Nitric oxide synthase, inducible	NOS2	3.38	1	1	1	1	6.234E7	1154	131.8	8.10
Q50KA9	Nucleoside diphosphate kinase A	NME1	20.39	1	2	2	2	3.082E7	152	17.2	6.01
Q95156	Olfactory receptor-like protein OLF3	N/A	12.93	1	1	1	1		317	35.2	8.22
Q8MJ04	Oxygen-regulated protein 1	RP1	1.12	1	1	1	1		2141	239.9	6.65
Q28256	Platelet glycoprotein Ib alpha chain	GP1BA	1.03	1	1	1	1	2.770E8	677	74.1	5.91
Q9GL24	Procathepsin L	CTSL	7.21	1	2	2	2	6.320E7	333	37.4	7.09
Q9XSR3	RNA-binding protein 47	RBM47	1.86	1	1	1	1	1.598E7	592	63.5	8.05
Q28263	Rod cGMP-specific 3',5'-cyclic phosphodiesterase subunit alpha	PDE6A	2.32	1	1	1	1	1.026E8	861	99.6	5.63
O46674	Sarcoplasmic/endoplasmic reticulum calcium ATPase 2	ATP2A2	1.20	1	1	1	1	1.100E7	997	109.6	5.36
P54832	Serum paraoxonase/arylesterase 2	PON2	5.08	1	1	1	1	8.273E6	354	39.6	6.09
Q6Y4Q5	Stromelysin-1	MMP3	3.77	1	1	1	1	3.853E7	478	53.6	5.48
A0M8U6	Testin		7.36	1	1	1	1	4.126E6	421	48.0	7.78
O62683	Tight junction protein ZO-3	TJP3	5.46	1	1	1	1		898	98.4	7.31
P54714	Triosephosphate isomerase	TPI1	24.10	1	3	3	3	7.346E7	249	26.7	7.33
P63050	Ubiquitin-60S ribosomal protein L40	UBA52	36.72	1	4	4	4	1.013E8	128	14.7	9.83
Q9N1T2	X-linked retinitis pigmentosa GTPase regulator	RPGR	1.00	1	1	1	1		1003	111.0	4.93

Q5QQ50	Xylosyltransferase 2	XYLT2	2.43	1	1	1	1	7.679E8	865	96.7	8.40
Q5TJF6	Zinc transporter SLC39A7	SLC39A7	4.26	1	1	1	1		469	49.9	6.95

1: Peptide spectrum matches

2: Amino acids

3: Molecular weight

4: calculated isoeletric point

5: E*n* is equivalent to 10^n.

Table S5. Proteins identified exclusively in semen sperm-rich fraction or in semen prostatic fraction from male dogs (protein identification by LC-MS/MS).

Accession No.	Description name
	Proteins identified exclusively in sperm-rich fraction (*n* = 37)
E2RKA8	60S ribosomal protein L32
Q6W3F4	Adenosine receptor A2b
Q28343	Aggrecan core protein
Q5F2N4	Alpha-(1,3)-fucosyltransferase 10
P58027	Amine oxidase [flavin-containing] A
P61797	Aprataxin
Q45T69	Bcl-2-like protein 2
Q5TJG6	Bromodomain-containing protein 2
F1PAA9	Cadherin-1
Q5ECR9	C-C chemokine receptor type 5
O18806	Coagulation factor VIII
F1PA44	Coiled-coil domain containing protein 112 (Submitted name)
Q5U820	Cystic fibrosis transmembrane conductance regulator
Q2KNA0	Cytospin-A
Q767J3	Deoxyribonuclease-1
P13206	Endothelin-1
Q64HP0	G1/S-specific cyclin-D1
P30552	Gastrin/cholecystokinin type B receptor
Q4PRC2	Glutamate decarboxylase 2
P17124	Histamine H2 receptor
Q29467	Inositol polyphosphate-5-phosphatase A
Q28268	Interleukin-12 subunit beta
Q1ZZS1	Lengsin
Q30DN6	Lysine-specific demethylase 5D
F1P660	Mediator complex subunit 23 (Submitted name)
F1P7W1	Olfactory receptor
F1PH13	Potassium channel tetramerization domain containing 8 (Submitted name)
Q6Q7P4	Protein 4.1
E2QY99	Protein PALS1
Q5XM32	Relaxin receptor 2
Q75W64	Single-stranded DNA cytosine deaminase
P23685	Sodium/calcium exchanger 1
Q9TTW3	Tektin-1
A8C750	Thyroid adenoma-associated protein homolog
Q8HYB7	Thyroid peroxidase
Q5I2M8	Toll-like receptor 9
F1PAB2	Transport and Golgi organization 6 homolog (Submitted name)
	Proteins identified exclusively in prostatic fraction (*n* = 29)
Q6XXX9	5-hydroxytryptamine receptor 1A
Q5TJE9	40S ribosomal protein S18
D0VWQ5	60S ribosomal protein L19
P79143	Aminopeptidase N
Q7YRF1	AP-3 complex subunit beta-1
E2RE76	Apolipoprotein A-IV
P49819	Dihydrolipoyl dehydrogenase, mitochondrial
E2RQ08	Dolichyl-diphosphooligosaccharide--protein glycosyltransferase subunit 1
Q4VSN4	Dual serine/threonine and tyrosine protein kinase

P62872	Guanine nucleotide-binding protein G(I)/G(S)/G(T) subunit beta-1
Q28270	Hematopoietic progenitor cell antigen
P39881	Homeobox protein cut-like 1 (Fragment)
Q00M95	Inactive rhomboid protein 2
Q076A6	Myosin-1
Q258K2	Myosin-9
Q50KA9	Nucleoside diphosphate kinase A
Q95156	Olfactory receptor-like protein OLF3
Q8MJ04	Oxygen-regulated protein 1
Q28256	Platelet glycoprotein Ib alpha chain
Q9GL24	Procathepsin L
Q9XSR3	RNA-binding protein 47
Q28263	Rod cGMP-specific 3',5'-cyclic phosphodiesterase subunit alpha
O46674	Sarcoplasmic/endoplasmic reticulum calcium ATPase 2
Q6Y4Q5	Stromelysin-1
A0M8U6	Testin
P54714	Triosephosphate isomerase
Q9N1T2	X-linked retinitis pigmentosa GTPase regulator
Q5QQ50	Xylosyltransferase 2
Q5TJF6	Zinc transporter SLC39A7

Table S6. Biological processes in which proteins detected in blood plasma, semen prostatic fraction and semen sperm-rich fraction from male dogs, were involved (protein identification by LC-MS/MS).

Biological processes	Proteins identified in		
	blood plasma (*n* =59)	sperm-rich fraction (*n* = 42)	prostatic fraction (*n* = 43)
Blood coagulation and haemostasis	1	1	1
Cell communication	4	5	6
Cell cycle	11	9	5
Cell death - Apoptosis	2	2	1
Cell organization and biogenesis	8	7	9
Glycolytic process	1	1	2
Inflammatory and defence response	5	4	3
Metabolic processes	10	5	5
Stress response	1	0	1
Transport of ions and molecules	12	3	6
Other	3	3	2
No annotation available	1	2	2

Table S7. Subcellular location of proteins detected in blood plasma, semen prostatic fraction and semen sperm-rich fraction from male dogs (protein identification by LC-MS/MS).

Subcellular location	Proteins identified in		
	blood plasma (*n* =59)	sperm-rich fraction (*n* = 42)	prostatic fraction (*n* = 43)
Cell membrane	14	15	15
Cytoplasm	23	8	16
Mitochondria	1	2	1
Nucleus	10	7	7
Secreted	10	6	4
No annotation available	1	4	0

Table S8. Molecular function of proteins detected in blood plasma, semen prostatic fraction and semen sperm-rich fraction from male dogs (protein identification by LC-MS/MS).

Molecular function	Proteins identified in		
	blood plasma (*n* =59)	sperm-rich fraction (*n* = 42)	prostatic fraction (*n* = 43)
Binding	22	11	17
Enzyme regulatory activity	18	11	15
Receptors	5	7	2
Structural molecular activity	4	1	2
Other	6	4	4
No annotation available	4	8	3

Table S9. Details of the biological process, the subcellular location and the molecular function of all proteins(n = 128) identified in the study (protein identification by LC-MS/MS) (in alphabetical order).

Accession No	Accession Name	Description Name	Tissue in which Identified [1]	Biological Process in which Involved	Subcellular Location	Molecular Function
Q5TJF5	DHB8_CANLF	(3R)-3-hydroxyacyl-CoA dehydrogenase	BP	Metabolic process	Mitochondria	Enzyme regulatory activity
Q5TJE9	RS18_CANLF	40S ribosomal protein S18	PF	Cell cycle	Cytoplasm	Binding
Q6XXX9	5HT1A_CANLF	5-hydroxytryptamine receptor 1A	PF	Cell communication	Cell membrane	Receptors
E2QXF3	RL15_CANFA	60S ribosomal protein L15	BP	Cell cycle	Cell membrane	Structural molecular activity
D0VWQ5	RL19_CANLF	60S ribosomal protein L19	PF	Cell cycle	Nucleus	Structural molecular activity
E2RKA8	RL32_CANLF	60S ribosomal protein L32	SRF	Cell cycle	Cytoplasm	Structural molecular activity
P62286	ASPM_CANLF	Abnormal spindle-like microcephaly-associated protein homolog	BP, SRF	Cell cycle	Nucleus	Binding
Q8HY67	AN32A_CANLF	Acidic leucine-rich nuclear phosphoprotein 32 family member A	BP	Cell cycle	Nucleus	Binding
O18840	ACTB_CANLF	Actin, cytoplasmic 1	BP, PF	Cell organization and biogenesis	Cytoplasm	Binding
Q6W3F4	AA2BR_CANLF	Adenosine receptor A2b	SRF	no annotation available	Cell membrane	Receptors
Q28343	PGCA_CANLF	Aggrecan core protein	SRF	Cell organization and biogenesis	Secreted	Binding
Q5F2N4	FUT10_CANLF	Alpha-(1,3)-fucosyltransferase 10	SRF	Glycolytic process	Cytoplasm	Enzyme regulatory activity
P58027	AOFA_CANLF	Amine oxidase [flavin-containing] A	SRF	Metabolic process	Mitochondria	Enzyme regulatory activity
P79143	AMPN_CANLF	Aminopeptidase N	PF	Cell organization and biogenesis	Cell membrane	Enzyme regulatory activity
Q29471	ANX13_CANLF	Annexin A13	BP	Transport of ions and molecules	Cell membrane	Binding
Q7YRF1	AP3B1_CANLF	AP-3 complex subunit beta-1	PF	Transport of ions and molecules	Cytoplasm	no annotation available
P02648	APOA1_CANLF	Apolipoprotein A-I	BP	Metabolic process	Secreted	Binding

E2RE76	APOA4_CANLF	Apolipoprotein A-IV	PF	Transport of ions and molecules	Secreted	Binding
P18649	APOE_CANLF	Apolipoprotein E	PF, SRF	Transport of ions and molecules	Secreted	Binding
P61797	APTX_CANLF	Aprataxin	SRF	Cell cycle	Nucleus	Binding
P09582	ESTA_CANFA	Arginine esterase	BP, PF	Other	Cytoplasm	Enzyme regulatory activity
Q32KH9	ARSG_CANLF	Arylsulfatase G	BP	Metabolic process	Cytoplasm	Enzyme regulatory activity
A1E2V0	BIRC3_CANLF	Baculoviral IAP repeat-containing protein 3	BP	Inflammatory and defence responce	Cytoplasm	Enzyme regulatory activity
Q45T69	B2CL2_CANLF	Bcl-2-like protein 2	SRF	Cell death - Apoptosis	Mitochondria	Binding
Q5TJE8	B3GT4_CANLF	Beta-1,3-galactosyltransferase 4	BP	Metabolic process	Cytoplasm	Enzyme regulatory activity
Q95153	BRCA1_CANLF	Breast cancer type 1 susceptibility protein homolog	BP	Cell cycle	Nucleus	Binding
Q5TJG6	BRD2_CANLF	Bromodomain-containing protein 2	SRF	Cell cycle	Nucleus	Other
F1PAA9	CADH1_CANLF	Cadherin-1	SRF	Cell communication	Cell membrane	Binding
E2RA18	CLGN_CANLF	Calmegin	BP	Transport of ions and molecules	Cytoplasm	Other
P24643	CALX_CANLF	Calnexin	BP	Cell organization and biogenesis	Cytoplasm	Other
Q9GL24	CATL1_CANLF	Cathepsin L1	PF	Inflammatory and defence responce	Cell membrane	Enzyme regulatory activity
Q5ECR9	CCR5_CANLF	C-C chemokine receptor type 5	SRF	Inflammatory and defence responce	Cell membrane	Receptors
O77746	PDE5A_CANLF	cGMP-specific 3',5'-cyclic phosphodiesterase	BP	Metabolic process	Cytoplasm	Enzyme regulatory activity
P25473	CLUS_CANLF	Clusterin	BP, PF	Cell death - Apoptosis	Cytoplasm	Other
O18806	FA8_CANLF	Coagulation factor VIII	SRF	Blood coagulation and haemostasis	Secreted	Enzyme regulatory activity
E2R1I5	CCD39_CANFA	Coiled-coil domain-containing protein 39	BP, PF	Other	Cytoplasm	no annotation available
F1PA44	F1PA44_CANFA	Coiled-coil domain containing protein 112 (Submitted name)	SRF	no annotation available	no annotation available	no annotation available

Q3LSL6	CXCR4_CANLF	C-X-C chemokine receptor type 4	BP	Cell organization and biogenesis	Cell membrane	Receptors
Q5U820	CFTR_CANLF	Cystic fibrosis transmembrane conductance regulator	SRF	Transport of ions and molecules	Cell membrane	Binding
Q2KNA0	CYTSA_CANLF	Cytospin-A	SRF	Cell cycle	Cytoplasm	no annotation available
Q767J3	DNAS1_CANLF	Deoxyribonuclease-1	SRF	Cell death - Apoptosis	Secreted	Enzyme regulatory activity
P49819	DLDH_CANLF	Dihydrolipoyl dehydrogenase, mitochondrial	PF	Metabolic process	Mitochondria	Enzyme regulatory activity
Q8WN22	PRKDC_CANLF	DNA-dependent protein kinase catalytic subunit	BP, PF	Inflammatory and defence responce	Nucleus	Enzyme regulatory activity
E2RQ08	RPN1_CANLF	Dolichyl-diphosphooligosaccharide--protein glycosyltransferase subunit 1	PF	Glycolytic process	Cell membrane	no annotation available
Q4VSN4	DUSTY_CANLF	Dual serine/threonine and tyrosine protein kinase	PF	Cell organization and biogenesis	Cell membrane	Enzyme regulatory activity
P41148	ENPL_CANLF	Endoplasmin	BP	Transport of ions and molecules	Cytoplasm	Other
P13206	EDN1_CANLF	Endothelin-1	SRF	Cell organization and biogenesis	Secreted	Other
Q9GL25	ESPB1_CANLF	Epididymal sperm-binding protein 1	BP	Cell organization and biogenesis	Secreted	Binding
P68213	FIBA_CANLF	Fibrinogen alpha chain	BP	Inflammatory and defence responce	Secreted	no annotation available
P02677	FIBB_CANLF	Fibrinogen beta chain (Fragment)	BP	Inflammatory and defence responce	Secreted	no annotation available
Q64HP0	CCND1_CANLF	G1/S-specific cyclin-D1	SRF	Cell cycle	Nucleus	Other
Q659K0	CCNB3_CANFA	G2/mitotic-specific cyclin-B3	BP, PF	Cell cycle	Nucleus	Binding
A2IBY7	CRYGS_CANLF	Gamma-crystallin S	BP	Other	no annotation available	Structural molecular activity
P30552	GASR_CANLF	Gastrin/cholecystokinin type B receptor	SRF	Other	Cell membrane	Receptors
A0PA85	DCE1_CANFA	Glutamate decarboxylase 1	BP	Metabolic process	Cell membrane	Enzyme regulatory activity
Q4PRC2	DCE2_CANLF	Glutamate decarboxylase 2	SRF	Metabolic process	Cell membrane	Enzyme regulatory activity

Q28259	G3P_CANLF	Glyceraldehyde-3-phosphate dehydrogenase	BP, PF	Metabolic process	Nucleus	Enzyme regulatory activity
P62872	GBB1_CANLF	Guanine nucleotide-binding protein G(I)/G(S)/G(T) subunit beta-1	PF	Cell organization and biogenesis	Cell membrane	Binding
Q7YQC6	HSP71_CANLF	Heat shock 70 kDa protein 1	BP, PF	Stress response	Cytoplasm	Other
Q28270	CD34_CANFA	Hematopoietic progenitor cell antigen	PF	Cell communication	Cell membrane	Binding
P60529	HBA_CANLF	Hemoglobin subunit alpha	BP	Transport of ions and molecules	Cytoplasm	Binding
P60524	HBB_CANLF	Hemoglobin subunit beta	BP	Transport of ions and molecules	Cytoplasm	Binding
P17124	HRH2_CANLF	Histamine H2 receptor	SRF	Cell communication	Cell membrane	Receptors
P39881	CUX1_CANFA	Homeobox protein cut-like 1 (Fragment)	PF	Cell cycle	Nucleus	Binding
P01784	HV01_CANLF	Ig heavy chain V region GOM	BP	Inflammatory and defence responce	Secreted	Binding
Q00M95	RHDF2_CANFA	Inactive rhomboid protein 2	PF	Transport of ions and molecules	Cell membrane	Binding
Q29467	I5P1_CANLF	Inositol polyphosphate-5-phosphatase A	SRF	Cell communication	Cell membrane	Enzyme regulatory activity
Q28268	IL12B_CANLF	Interleukin-12 subunit beta	SRF	Inflammatory and defence responce	Secreted	Other
Q95LF0	I13R2_CANLF	Interleukin-13 receptor subunit alpha-2	BP	Cell communication	Cell membrane	Receptors
Q6EIY9	K2C1_CANLF	Keratin, type II cytoskeletal 1	PF, SRF	Cell organization and biogenesis	Cell membrane	Binding
Q1ZZS1	LGSN_CANLF	Lengsin	SRF	Metabolic process	no annotation available	Enzyme regulatory activity
Q5XNR9	LIFR_CANLF	Leukemia inhibitory factor receptor	BP	Cell communication	Cell membrane	Receptors
Q30DN6	KDM5D_CANLF	Lysine-specific demethylase 5D	SRF	Cell organization and biogenesis	Nucleus	Enzyme regulatory activity
E2QY99	PALS1_CANLF	MAGUK p55 subfamily member 5	SRF	Cell organization and biogenesis	Cell membrane	Binding
F1P660	F1P660_CANFA	Mediator complex subunit 23 (Submitted name)	SRF	Cell cycle	Nucleus	no annotation available

P35521	ICLN_CANLF	Methylosome subunit pICln	BP	Cell cycle	Nucleus	Binding
P23294	MYPR_CANLF	Myelin proteolipid protein	BP	Cell organization and biogenesis	Cell membrane	Structural molecular activity
Q076A6	MYH1_CANFA	Myosin-1	PF	no annotation available	Cytoplasm	Binding
P49824	MYH7_CANLF	Myosin-7	BP	Metabolic process	Cytoplasm	Binding
Q258K2	MYH9_CANLF	Myosin-9	PF	Cell communication	Cytoplasm	Binding
Q9TU19	NPHP1_CANLF	Nephrocystin-1 (Fragment)	BP, SRF	Cell organization and biogenesis	Cytoplasm	no annotation available
O02813	NPY1R_CANLF	Neuropeptide Y receptor type 1	BP	Metabolic process	Cell membrane	Receptors
O62699	NOS2_CANLF	Nitric oxide synthase, inducible	PF, SRF	Inflammatory and defence responce	Cytoplasm	Enzyme regulatory activity
Q50KA9	NDKA_CANLF	Nucleoside diphosphate kinase A	PF	Cell organization and biogenesis	Nucleus	Enzyme regulatory activity
F1P7W1	F1P7W1_CANFA	Olfactory receptor	SRF	Cell communication	Cell membrane	Receptors
Q95156	OLF3_CANLF	Olfactory receptor-like protein OLF3	PF	Cell communication	Cell membrane	Receptors
Q8MJ04	RP1_CANLF	Oxygen-regulated protein 1	PF	Cell organization and biogenesis	Cytoplasm	Binding
Q0ZAQ8	PPARD_CANLF	Peroxisome proliferator-activated receptor delta	BP	Cell cycle	Nucleus	Binding
P80009	PLMN_CANLF	Plasminogen (Fragment)	BP	Blood coagulation and haemostasis	Secreted	Enzyme regulatory activity
Q28256	GP1BA_CANFA	Platelet glycoprotein Ib alpha chain	PF	Blood coagulation and haemostasis	Cell membrane	Binding
Q6Q308	PLEK_CANLF	Pleckstrin	BP	Cell organization and biogenesis	Cell membrane	Enzyme regulatory activity
F1PH13	F1PH13_CANFA	Potassium channel tetramerization domain containing 8 (Submitted name)	SRF	Cell organization and biogenesis	Cell membrane	no annotation available
Q9GLW0	PRGR_CANLF	Progesterone receptor	BP	Cell cycle	Cytoplasm	Receptors
Q5XXR2	MCH_CANLF	Pro-MCH	BP	Cell communication	Secreted	Other
Q6Q7P4	41_CANLF	Protein 4.1	SRF	Cell cycle	Cytoplasm	Binding
Q5PU49	KPCD_CANLF	Protein kinase C delta type	BP	Cell death - Apoptosis	Cytoplasm	Enzyme regulatory activity
Q29536	KPYR_CANLF	Pyruvate kinase	BP	Glycolytic process	Cytoplasm	Enzyme regulatory activity

P24409	RAB10_CANLF	Ras-related protein Rab-10	BP	Transport of ions and molecules	Cytoplasm	Enzyme regulatory activity
P61105	RAB2A_CANLF	Ras-related protein Rab-2A	BP	Transport of ions and molecules	Secreted	Binding
Q5XM32	RXFP2_CANLF	Relaxin receptor 2	SRF	Cell communication	Cell membrane	Receptors
Q9XSR3	RBM47_CANLF	RNA-binding protein 47	PF	Metabolic process	Nucleus	Binding
Q28263	PDE6A_CANFA	Rod cGMP-specific 3',5'-cyclic phosphodiesterase subunit alpha	PF	Cell communication	Cell membrane	Enzyme regulatory activity
O46674	AT2A2_CANLF	Sarcoplasmic/endoplasmic reticulum calcium ATPase 2	PF	Transport of ions and molecules	Cytoplasm	Enzyme regulatory activity
P06871	TRY1_CANLF	Serine Protease 1	BP	Metabolic process	Secreted	Enzyme regulatory activity
P49822	ALBU_CANLF	Serum albumin	BP	Transport of ions and molecules	Secreted	Binding
P54832	PON2_CANFA	Serum paraoxonase/arylesterase 2	BP, PF	no annotation available	Cell membrane	Enzyme regulatory activity
P61010	SRP54_CANLF	Signal recognition particle 54 kDa protein	BP	Cell communication	Cytoplasm	Binding
Q75W64	AICDA_CANLF	Single-stranded DNA cytosine deaminase	SRF	Cell cycle	Nucleus	Enzyme regulatory activity
P23685	NAC1_CANLF	Sodium/calcium exchanger 1	SRF	Transport of ions and molecules	Cell membrane	Binding
P50997	AT1A1_CANLF	Sodium/potassium-transporting ATPase subunit alpha-1	BP	Transport of ions and molecules	Cell membrane	Enzyme regulatory activity
Q5WR10	DX39B_CANLF	Spliceosome RNA helicase DDX39B	BP	Transport of ions and molecules	Nucleus	Enzyme regulatory activity
Q6Y4Q5	MMP3_CANFA	Stromelysin-1	PF	Metabolic process	Secreted	Enzyme regulatory activity
Q9TTW3	TEKT1_CANLF	Tektin-1	SRF	Other	Cytoplasm	no annotation available
A0M8U6	TES_CANLF	Testin	PF	Cell organization and biogenesis	Cytoplasm	Binding
A8C750	THADA_CANLF	Thyroid adenoma-associated protein homolog	SRF	Metabolic process	no annotation available	no annotation available
Q8HYB7	PERT_CANLF	Thyroid peroxidase	SRF	Metabolic process	Cell membrane	Enzyme regulatory activity

O62683	ZO3_CANLF	Tight junction protein ZO-3	BP, PF	Cell communication	Cell membrane	Binding
Q5I2M8	TLR9_CANLF	Toll-like receptor 9	SRF	Inflammatory and defence responce	Cytoplasm	Receptors
Q697L1	TRPV1_CANLF	Transient receptor potential cation channel subfamily V member 1	BP	Transport of ions and molecules	Cell membrane	Binding
F1PAB2	F1PAB2_CANFA	Transport and Golgi organization 6 homolog (Submitted name)	SRF	Other	no annotation available	no annotation available
P54714	TPIS_CANLF	Triosephosphate isomerase	PF	Glycolytic process	Cytoplasm	Enzyme regulatory activity
E2RDV1	TDRD7_CANLF	Tudor domain-containing protein 7	BP	Cell cycle	Cytoplasm	Binding
P63050	RL40_CANLF	Ubiquitin-60S ribosomal protein L40	BP, PF	Cell cycle	Cytoplasm	Structural molecular activity
Q5TJF0	VPS52_CANLF	Vacuolar protein sorting-associated protein 52 homolog	BP	Transport of ions and molecules	Cell membrane	Binding
Q9N1T2	RPGR_CANFA	X-linked retinitis pigmentosa GTPase regulator	PF	Cell organization and biogenesis	Cytoplasm	Other
Q5QQ50	XYLT2_CANLF	Xylosyltransferase 2	PF	Metabolic process	Secreted	Enzyme regulatory activity
Q9XSR1	ZN252_CANLF	Zinc finger protein	BP	Cell cycle	Nucleus	Binding
Q5TJF6	S39A7_CANLF	Zinc transporter SLC39A7	PF	Transport of ions and molecules	Cell membrane	Other

1: BP: blood plasma, PF: prostatic fraction, SRF: sperm-rich fraction.
